# The Impact of Polymorphisms in ATP-Binding Cassette Transporter Genes on Anthracycline-Induced Early Cardiotoxicity in Patients with Breast Cancer

**DOI:** 10.3390/jcdd10060232

**Published:** 2023-05-26

**Authors:** Gintare Muckiene, Domas Vaitiekus, Diana Zaliaduonyte, Agne Bartnykaite, Jurgita Plisiene, Vytautas Zabiela, Elona Juozaityte, Renaldas Jurkevicius

**Affiliations:** 1 Cardiology Clinic, Medical Academy, Lithuanian University of Health Sciences, LT-44307 Kaunas, Lithuania; 2 Department of Cardiology, Hospital of Lithuanian University of Health Sciences, LT-50161 Kaunas, Lithuania; 3 Kaunas Region Society of Cardiology, LT-44307 Kaunas, Lithuania; 4 Oncology Institute, Lithuanian University of Health Sciences, LT-50161 Kaunas, Lithuania; 5 Department of Oncology and Hematology, Hospital of Lithuanian University of Health Sciences, LT-50161 Kaunas, Lithuania; 6 Cardiology Department, Kaunas Hospital of Lithuanian University of Health Sciences, LT-47144 Kaunas, Lithuania; 7 Institute of Cardiology, Lithuanian University of Health Sciences, LT-50161 Kaunas, Lithuania

**Keywords:** anthracycline, doxorubicin, breast cancer, cardiotoxicity, ATP-binding cassette transporters, polymorphism

## Abstract

Background. Cardiac side effects associated with anthracycline-based treatment may seriously compromise the prognosis of patients with breast cancer (BC). Evidence shows that genes that operate in drug metabolism can influence the risk of anthracycline-induced cardiotoxicity (AIC). ATP-binding cassette (ABC) transporters could serve as one of the potential biomarkers for AIC risk stratification. We aimed to determine the link between single-nucleotide polymorphisms (SNPs) in several *ABC* genes (*ABCB1* rs1045642, *ABCC1* rs4148350, *ABCC1* rs3743527) and cardiotoxicity. Methods. The study included 71 patients with BC, who were treated with doxorubicin-based chemotherapy. Two-dimensional echocardiography and speckle-tracking echocardiography were performed. AIC was defined as a new decrease of 10 percentage points in the left ventricular ejection fraction (LVEF). SNPs in *ABCB1* and *ABCC1* genes were evaluated using real-time PCR. Results. After a cumulative dose of 236.70 mg/m^2^ of doxorubicin, 28.2% patients met the criteria of AIC. Patients who developed AIC had a larger impairment in left ventricular systolic function compared to those who did not develop AIC (LVEF: 50.20 ± 2.38% vs. 55.41 ± 1.13%, *p* < 0.001; global longitudinal strain: −17.03 ± 0.52% vs. −18.40 ± 0.88%, *p* < 0.001). The *ABCC1* rs4148350 TG genotype was associated with higher rates of cardiotoxicity (TG vs. GG OR = 8.000, 95% CI = 1.405–45.547, *p* = 0.019). Conclusions. The study showed that *ABCC1* rs4148350 is associated with AIC and could be a potential biomarker to assess the risk of treatment side effects in patients with BC.

## 1. Introduction

In recent years, the progress of cancer therapy has been surprising. Advances in treatment have led to improved survival of patients with cancer but have also increased morbidity and mortality due to treatment side effects [1,2].

Anthracyclines are commonly used chemotherapy agents in the treatment of various malignancies. Doxorubicin is an anthracycline antibiotic that was discovered from a mutated strain of *Streptomyces peucetius*. It belongs to the anthracycline family and is currently the most effective chemotherapeutic drug widely used to treat breast cancer [3]. Moreover, there are a number of studies focusing on clarifying the pathways related to doxorubicin, finding an effective way for making doxorubicin-based chemotherapy more tolerable, and inhibiting drug resistance in breast cancer cell cultures and several animal models [4,5,6,7,8,9].

Guidelines published by the American Association of Clinical Oncology (ASCO) state that cardiac dysfunction, as one of the most common adverse events in clinical practice in the treatment of oncological patients, has adverse effects on treatment efficacy, quality of life, and survival [10]. Cardiac side effects associated with anthracycline-based treatment may seriously compromise the prognosis and well-being of patients with breast cancer.

Cancer-therapeutic-related cardiac dysfunction is defined as a decrease of 10 percentage points in the left ventricular ejection fraction (LVEF) to a value below the lower limit of normal (LVEF < 53%). Furthermore, a relative percentage reduction of 15% in the global longitudinal strain (GLS) from baseline is considered abnormal and may be evaluated as a marker of early left ventricular (LV) subclinical dysfunction [11,12].

Anthracycline-induced cardiotoxicity (AIC) can manifest as asymptomatic LV dysfunction in up to 57% of treated patients [13,14] and cardiomyopathy resulting in congestive heart failure (HF) in up to 16–20% of patients [15,16].

Despite extensive research, the pathogenetic mechanisms responsible for AIC have not been fully elucidated. It is known that a cumulative dose is the greatest risk factor for developing doxorubicin-induced cardiotoxicity. A dose of over 450 mg/m^2^ of doxorubicin during a lifetime substantially increases the risk of clinically significant HF [17]. However, there are numerous other risk factors for AIC according to guidelines and other publications [11,12,18]. Therefore, a primary goal for cardiologists and oncologists should be the early identification of patients at risk of cardiotoxicity.

The genetic predisposition for cardiotoxicity has been discussed widely. Recognition of the genetic predisposition of an individual patient’s susceptibility to the cardiotoxic effects of anthracycline could improve the safety of doxorubicin-based treatment. The search for genetic factors in AIC includes genes that regulate drug transport.

If mutations that increase the risk of doxorubicin-induced cardiotoxicity are known, patients with those mutations can be identified at the time of diagnosis and treated with cardioprotective medications to lower their risk of developing HF. Therefore, early detection of individuals at risk of cardiotoxicity should be a top priority for cardiologists and oncologists.

ABC transporters, or ATP-binding cassette transporters, are a large family of transmembrane macromolecules responsible for the uptake or export of substitutes by using ATP energy across cell membranes. It is known that this family of proteins has an impact on the pharmacokinetics of drugs and may also influence drug resistance [19,20]. The relationship between the expression of ABC transporters and the different effects of anticancer drugs, such as anthracyclines, was detected in various cancer cells [21]. For example, a study on breast cancer cell lines demonstrated that the efflux of doxorubicin is dependent on the activity of ABCB4 [22]. Wen et al. showed that the sensitivity of breast cancer cells to doxorubicin can be increased by downregulating the expression of ABCB1, ABCB5, and ABCG2 [23]. In a study of Gatta et al., it was demonstrated that breast cancer cells can be sensitized to doxorubicin by reducing ABCC1 expression [24]. Additionally, ABCC1 is an important factor that regulates doxorubicin transport in lung and prostate cancer cells [25,26], while ABCB1 regulates the sensitivity for doxorubicin in colon cancer cells [27]. ABCG2 regulated the impact of doxorubicin transport in mice xenograft models [28]. Many ABC transporters tend to be related to chemotherapeutic drug pathways; therefore, more studies should be performed to get a better understanding of the precise mechanisms and elucidate the genetic factors that may influence ABC transporter functions.

However, few studies have focused on the association between the genetic variants of *ABC* genes and the risk of doxorubicin-induced cardiotoxicity in breast cancer. In this study, we aimed to determine the link between *ABCB1* rs1045642, *ABCC1* rs4148350, and *ABCC1* rs3743527 and AIC.

## 2. Materials and Methods

### 2.1. Study Population

**Figure 1:**
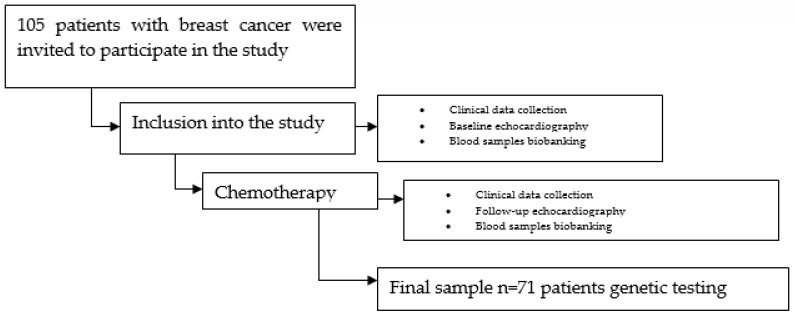
A flowchart of patient selection.

This prospective study was performed at the Cardiology Department and the Oncology and Hematology Department at the Hospital of Lithuanian University of Health Sciences Kauno Klinikos. A total of 105 patients with breast cancer, who were treated with doxorubicin-based chemotherapy, were invited to participate in the study. Of these, 34 patients were not included due to the exclusion criteria or because they later declined to participate. The final analytic cohort included 71 patients. A flowchart of patient selection is shown in Figure 1.

Inclusion criteria for this study were:

(1) Women aged >18 years, (2) a diagnosis of stage I–III of breast cancer (non-metastatic disease), (3) qualification for chemotherapy regimens with conventional doxorubicin, (4) normal systolic LV function on echocardiography (baseline LV ejection fraction (LVEF) ≥ 55%) and no typical signs of HF before the onset of anticancer treatment, and (5) written informed consent to participate in the study.

Exclusion criteria for this study were:

(1) Previous radiation therapy involving the heart and previous chemotherapy; (2) known significant LV and right ventricular (RV) dysfunction, severe valvular heart disease, arrhythmias, or mental illness; (3) contraindication for doxorubicin-based chemotherapy (including significant renal failure: glomerular filtration rate (GFR) < 30 mL/min; other significant medical contraindications according to the treating medical oncologist); (4) poor-quality echocardiography windows; (5) patient’s refusal to participate in the study; and (6) single-nucleotide polymorphism (SNP) genotyping failure due to non-amplification.

The distribution of chemotherapy regimens:

- AC (doxorubicin 60 mg/m^2^ + cyclophosphamide 600 mg/m^2^ every 3 weeks for four cycles);
- AC-P (doxorubicin 60 mg/m^2^ + cyclophosphamide 600 mg/m^2^ every 3 weeks for four cycles, followed by paclitaxel 80 mg/m^2^ weekly for 12 times or paclitaxel 175 mg/m^2^ every 21 days for four cycles);
- AC + D (doxorubicin 60 mg/m^2^ + cyclophosphamide 600 mg/m^2^ every 3 weeks for four cycles, followed by docetaxel 100 mg/m^2^ every 3 weeks for four cycles);
- FAC + D (5-FU 500 mg/m^2^ on days 1 and 8 + doxorubicin 50 mg/m^2^ on day 1 + cyclophosphamide 500 mg/m^2^ on day 1 every 3 weeks for three cycles, followed by docetaxel 100 mg/m^2^ every 3 weeks for three cycles);
- TAC (docetaxel 75 mg/m^2^ + doxorubicin 50 mg/m^2^ + cyclophosphamide 500 mg/m^2^ IV every 3 weeks for six cycles);
- FAC (5-FU 500 mg/m^2^ on days 1 and 8 + doxorubicin 50 mg/m^2^ on day 1 + cyclophosphamide 500 mg/m^2^ on day 1 every 3 weeks for six cycles).

In all regimens, doxorubicin was administered using 1 h intravenous infusion. Trastuzumab was administered with taxanes and later as monotherapy after doxorubicin standard of care in an adjuvant setting for a 52-week duration. Trastuzumab use did not have a statistically significant influence on LVEF and/or GLS decrease (*p* > 0.05).

Permission for this prospective research was obtained from the Kaunas Regional Biomedical Research Bioethics Committee (ref. no. P1-BE-2-10). The study was conducted in accordance with the Declaration of Helsinki. All patients signed an informed consent form.

### 2.2. Echocardiography

All patients underwent 2D transthoracic echocardiography and strain imaging with 2D speckle-tracking echocardiography (STE) before anthracycline-based treatment was initiated and after completion of four cycles of chemotherapy. All images were obtained by one investigator with an ultrasonography system (EPIQ 7, Phillips Ultrasound, Inc., Washington, DC, USA) equipped with an S_5-1_ (1 to 5 MHz) fully sampled matrix transducer.

Standard 2D echocardiography was performed according to the recommendations of the American Society of Echocardiography [29]. The analysis of LV longitudinal strain was performed in apical four-chamber, two-chamber, and long-axis views [30]. The global values of the LV longitudinal peak systolic strain (GLS) were obtained from the average of 18 segments computed from the three apical views.

We defined AIC as a new decrease of 10 percentage points in the LVEF to a value below the lower limit of normal (LVEF < 53%) [12].

### 2.3. Genotyping Methods

The single-nucleotide polymorphisms (SNPs) of *ABCB1* and *ABCC1* genes were estimated using TaqMan SNP Genotyping Assays (Applied Biosystems, Foster City, CA, USA). Reactions were assembled into a 25 μL total volume and included 2 ng of DNA, 12.5 μL of TaqMan™ Universal Master Mix II no UNG (2×) (Applied Biosystems, Foster City, CA, USA), 1.25 μL of diluted 20× TaqMan SNP Genotyping Assay, and nuclease-free water. A no-template control (nuclease-free water instead of DNA) was used to confirm the lack of contamination in every run. The DNA of known genotypes was used as a positive control. The conditions of reactions were set as follows: heating at 95 °C for 10 min, followed by 40 cycles at 95 °C for 15 s and 60 °C for 1 min. The 7900HT Real-Time Polymerase Chain Reaction System (Applied Biosystems, Foster City, CA, USA) was used for detection. The echocardiographer was blinded to the results of the genetic testing. For SNP genotyping, all samples stored at the tissue bank laboratory were genotyped regardless of further follow-up events. Genotyping was performed at the end of the study.

### 2.4. Statistical Analysis

The evaluation of the Hardy–Weinberg equilibrium (HWE) was conducted by comparing the observed and expected frequencies of genotypes using the chi-square (χ2) test. Continuous variables were analyzed using Student’s *t*-test or the Mann–Whitney U test. The genotype frequencies of polymorphisms, the distributions of clinical characteristics, and chemotherapy regimens were compared between groups using the chi-square or Fisher’s exact test. The odds ratios (ORs) and 95% confidence intervals (95% CIs) were calculated to estimate associations between SNPs and cardiotoxicity. Multivariate analyses were adjusted for GLS and NT-proBNP parameters, and for GLS and NT-proBNP, and the cumulative doxorubicin dose. *p* < 0.05 indicated statistical significance. Statistical analysis was performed with IBM SPSS Statistics 20.0.

## 3. Results

A total of 71 women with pathologically confirmed breast cancer were enrolled in this study. The study population age ranged from 33 to 75 years (mean age: 53.76 ± 9.23). Patients’ baseline clinical characteristics are presented in Table 1. None of the patients had symptoms or signs of HF during the study period. The baseline average value of NT-proBNP was normal (91.96 ± 42.64 ng/L) in the study population.

The most common CVD risk factors were AH (43.7%), dyslipidemia (25.4%), and a family history of premature atherosclerotic CVD (23.9%). β-Blockers and angiotensin-converting enzyme inhibitors/angiotensin receptor blockers were most commonly used in our study population.

Most subjects were detected to be at pathological stage IIA (33.8%) or IA (29.6%).

The distribution of chemotherapy regimens is shown in Table 2. The majority of subjects (66.4% in the noncardioxicity group and 65.0% in the cardiotoxicity group) were treated with AC-paclitaxel. All regimens included similar cumulative doses of doxorubicin. The median cumulative doxorubicin dose was 236.70 mg/m^2^ (range 129.00–303.20).

All study subjects had normal LV systolic function (LVEF: 60.51 ± 1.89%, range 56–65; GLS: −20.96 ± 0.51%, range −20.1 to 22.6) before anthracycline-based chemotherapy. Baseline M-mode and Doppler echocardiography analysis showed normal longitudinal LV systolic function as well as LV diastolic function. The baseline 2D echocardiographic parameters are summarized in Table 3.

All patients underwent clinical evaluation: 2DE and strain analysis using STE after completion of four cycles of chemotherapy. As AIC was defined as a new decrease of 10 percentage points in the LVEF to a value below the lower limit of normal [12], the studied patients were divided into two groups: patients with cardiotoxicity and patients without cardiotoxicity. A total of 20 patients (28.2%) met the criteria of LV cardiotoxicity after anthracycline-based chemotherapy.

The levels of NT-proBNP were measured before and after anthracycline-based chemotherapy in this study, and these levels increased significantly during the monitoring period from 91.96 ± 42.64 ng/L (range 17.80–172.00) at baseline to 143.36 ± 58.18 ng/L (36.00–285.00) at the completion of chemotherapy (*p* < 0.001). Women who progressed to AIC had significantly higher baseline serum levels of NT-proBNP compared to patients without cardiotoxicity (109.01 ± 36.97 ng/L vs. 84.83 ± 43.21 ng/L, *p* = 0.042; Table 1). In addition, the increase in NT-proBNP concentrations was more pronounced during the follow-up periods in women who developed cardiotoxicity than in women without cardiotoxicity (177.47 ± 53.72 ng/L vs. 129.87 ± 54.76 ng/L, *p* = 0.003).

The baseline LVEF and GLS were within normal limits in both groups. M-mode and Doppler echocardiographic parameters did not differ between the two groups at baseline (Table 4).

Patients who developed AIC during follow-up had a significantly larger impairment in LV systolic function compared to those who did not develop AIC (LVEF: 50.20 ± 2.38% vs. 55.41 ± 1.13%, *p* < 0.001; GLS: −17.03 ± 0.52% vs. −18.40 ± 0.88%, *p* < 0.001).

Table 4 summarizes the echocardiographic parameters and their comparisons at baseline and follow-up in women who developed cardiotoxicity and in women who remained without cardiotoxicity.

**Figure 2:**
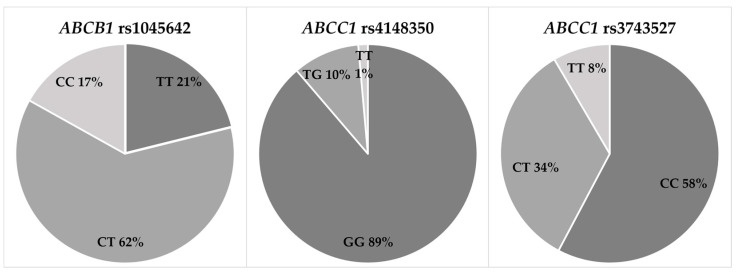
Genotype distribution of polymorphisms in the study group.

We aimed to determine the link between *ABCB1* rs1045642, *ABCC1* rs4148350, and *ABCC1* rs3743527 and cardiotoxicity in breast cancer. All observed genotype distributions, excluding *ABCB1* rs1045642 (*p* = 0.04), were in agreement with the Hardy–Weinberg equilibrium (*p* > 0.05). The genotype distributions of the studied SNPs are summarized in Figure 2. In this study, we found that the *ABCC1* rs4148350 TG genotype carriers experienced higher rates of cardiotoxicity than the GG genotype carriers (OR = 8.000, 95% CI = 1.405–45.547, *p* = 0.019). Additionally, similar results were found when comparing rs4148350 T allele carriers and non-carriers. The results remained statistically significant in both multivariate analyses: following adjustment for GLS and NT-proBNP (TG vs. GG: OR = 9.258, 95% CI = 1.364–62.846, *p* = 0.0023) and following adjustment for GLS, NT-proBNP, and the doxorubicin cumulative dose (TG vs. GG: OR = 9.661, 95% CI = 1.418–65.824, *p* = 0.0021). However, our results indicated that *ABCB1* rs1045642 and *ABCC1* rs3743527 did not influence the development of cardiotoxicity after anthracycline-based treatment in the studied group of patients with breast cancer (Table 5).

## 4. Discussion

Although improvements in cancer treatment have enhanced the survival of patient with cancer, they have also raised morbidity and mortality rates due to adverse effects. Anthracyclines have been in use since the 1960s. They are effective and widely used drugs in breast cancer treatment but have many side effects [31]. The most important side effect of anthracyclines is cardiotoxicity resulting in congestive HF [32].

AIC is a dose-dependent and cumulative process of variable onset that may present with symptomatic or asymptomatic cardiotoxicity [33]. This diagnosis is based on a reduction in the LVEF and relative changes in GLS [12].

AIC can manifest as asymptomatic LV dysfunction in up to 57% of treated patients [13,14]. In clinical practice, however, these percentages may be even higher. In our study, 28.2% of patients met the criteria of LV cardiotoxicity after chemotherapy.

AIC may occur through a variety of mechanisms, such as interaction with iron, the activity of intracellular or intramitochondrial oxidizing enzymes, alteration of endothelin-1 expression in cardiomyocytes, and binding to topoisomerases [14,32,33,34,35,36,37]. AIC is thought to be cumulative and dose dependent at a total doxorubicin dose of 450 mg/m^2^ [38]. Billingham et al. showed results of endomyocardial biopsies that revealed histopathological changes with doses as low as 240 mg/m^2^, suggesting that subclinical cardiotoxicity may be present as early as the first dose [39]. The median cumulative doxorubicin dose in our study was low too (236.70 mg/m^2^, range 129.0–303.2), and subclinical LV damage was also detected during our study.

Natriuretic peptides (B-type natriuretic peptide (BNP)/N-terminal pro-brain natriuretic peptide (NT-proBNP)) are biomarkers for cardiovascular disease (CVD) risk stratification and are widely used for the diagnosis of early AIC [40,41]. In our study, women who developed AIC had significantly higher baseline serum levels of NT-proBNP compared to those who did not develop AIC (109.01 ± 36.97 ng/L vs. 84.83 ± 43.21 ng/L, *p* = 0.042), suggesting that some of the study patients already had increased biological stress and myocardial strain [42] due to preexisting CV risk factors (e.g., arterial hypertension). Moreover, increased levels of NT-proBNP were observed following doxorubicin-based chemotherapy and higher NT-proBNP levels in the AIC group compared to the no-cardiotoxicity group, suggesting that the increase was induced by anthracycline-based treatment.

According to the data, genes involved in medication metabolism can also affect the occurrence of cardiovascular side effects following cancer treatments [43]. There are 48 known human *ATP-binding cassette (ABC) transporter* genes, which are positioned in different chromosomal locations and are responsible for various functions [44]. Studies have shown that the ABCB1 transporter is important for drug efflux and resistance to several unrelated drugs used in cancer chemotherapy [45]. Moreover, ABCC1 is widely expressed in various human tissues and is responsible for multidrug resistance [46,47].

Although ABCB1 and ABCC1 are two of the most studied and cancer-related ABC transporters, there is a lack of studies analyzing their effect on AIC in breast cancer. Several polymorphisms in *ABCB1* and *ABCC1* genes have previously been correlated with AIC, but more studies are needed to reach unified conclusions. The aim of our study was to identify genetic variants (rs1045642, rs4148350, and rs3743527) in *ABC* genes and determine their role in AIC.

According to a meta-analysis performed in 2017 by Leong et al., *ABCB1* rs1045642 is associated with a lower risk of developing AIC. This polymorphism has been previously reported to be significantly associated with systolic dysfunction (EF < 55%) [47]. Hertz et al. observed that *ABCB1* rs1045642 has an additive protective effect against cardiotoxicity (OR = 0.48, 95% CI = 0.23–1.00, *p* = 0.049), and the results remained nominally significant after correction for clinical covariates (*p* = 0.048) [48]. However, we did not find a significant association between *ABCB1* rs1045642 and cardiotoxicity. The results of univariate and multivariate logistic regression analyses showed the potential cardioprotective effect of the rs1045642 heterozygous genotype, but it did not reach statistical significance. A previous Rossi et al. analysis that included rs1045642 also did not detect a cardioprotective or cardiotoxic effect. In their study, rs1045642 was not associated with grade 2–4 cardiotoxicity in patients with diffuse large B-cell lymphoma treated with R-CHOP21 (TT genotype vs. CT + CC: OR = 1.16, 95% CI = 0.73–1.84, *p* = 0.515; CT + TT vs. CC: OR = 1.09, 95% CI = 0.69–1.73, *p* = 0.680) [49].

A meta-analysis by Leong et al. showed that *ABCC1* rs4148350 is associated with a higher risk of developing AIC [47]. A childhood cancer cohort-based study by Visscher et al. described that *ABCC1* rs4148350 is associated with AIC. Another study found that rs4148350 can be classified as a risk-increasing polymorphism (OR = 3.44, 95% CI = 1.65–7.15, *p* = 0.0012) [50]. In our study, we more frequently observed the presence of cardiotoxicity in patients with the rs4148350 TG genotype compared to the GG genotype. The results remained statistically significant following the adjustment for more confounding variables (GLS, NT-proBNP, cumulative doxorubicin dose) and indicated the rs4148350 SNP as an independent prognostic factor for AIC. However, in contrast to our findings, Hertz et al. provided evidence that rs4148350 had no impact on cardiotoxicity development, assuming additive genetic effects (*p* = 0.92) in 166 patients with breast cancer [48]. However, there is a lack of studies analyzing the effect of *ABCC1* rs4148350 on AIC in breast cancer. Consequently, to assess the relevance of these findings, additional studies with independent cohorts are necessary.

In this study, we did not find significant associations between *ABCC1* rs3743527 and cardiotoxicity. According to Liu et al., in a cohort of 388 patients with breast cancer who received adjuvant chemotherapy, *ABCC1* rs3743527 was not found to be one of the genetic factors closely related to the risk of cardiotoxicity [51]. Semsei et al. analyzed *ABCC1* rs3743527 in patients with childhood acute lymphoblastic leukemia. The authors found that the TT genotype group had significantly decreased left ventricular fractional shortening (LVFS) at the end of treatment compared to the CT group, contributing to influenced cardiac function after anthracycline treatment [52]. Another study performed on a pediatric (2–18 years old) population diagnosed with acute lymphoblastic leukemia revealed that the CT genotype was associated with a decreased risk of developing AIC (OR = 0.30, 95% CI = 0.09–0.91, *p* = 0.03) [52,53].

## 5. Conclusions

This study provided evidence that *ABCC1* rs4148350 significantly increases the risk of early AIC in patients with breast cancer. Further studies involving other single-nucleotide polymorphisms and more patients are necessary to examine the more precise impact of *ABC* genes on AIC in breast cancer. If it is proved that SNPs are reliable biomarkers of cardiotoxicity prediction, it would contribute to the prospects of treatment individualization.

## Figures and Tables

**Table 1:** Baseline clinical characteristics in patients with or without cardiotoxicity.

	All Patients (*n* = 71)	Noncardiotoxicity (*n* = 51, 71.8%)	Cardiotoxicity (*n* = 20, 28.2%)	*p*-Value
Age (years)	53.76 ± 9.23	53.94 ± 8.42	53.30 ± 11.25	0.868
BMI (kg/m^2^)	28.44 ± 6.04	28.39 ± 6.09	28.55 ± 6.08	0.928
CVD risk factors				
AH, n (%)	31 (43.7)	16 (31.4)	15 (75.0)	0.001
Diabetes mellitus, n (%)	11 (15.5)	8 (15.7)	3 (15.0)	1.000
Smoking, n (%)	16 (22.5)	10 (19.6)	6 (30.0)	0.360
Family history of CVD, n (%)	17 (23.9)	8 (15.7)	9 (45.0)	0.014
Dyslipidemia, n (%)	18 (25.4)	12 (23.5)	6 (30.0)	0.561
Medications				
ACE inhibitors/ARBs	19 (26.8)	11 (21.6)	8 (40.0)	0.141
β-Blockers	25 (35.2)	12 (23.5)	13 (65.0)	0.002
Diuretics	6 (8.5)	2 (3.9)	4 (20.0)	0.049
Calcium channel blockers	6 (8.5)	3 (5.9)	3 (15.0)	0.340
NT pro-BNP (ng/L)	91.96 ± 42.64	84.83 ± 43.21	109.01 ± 36.97	0.042
Pathological stage (pTNM)				0.0901
0	3 (4.2)	3 (5.9)	0 (0.0)	
IA	21 (29.6)	14 (27.5)	7 (35.0)	
IB	11 (15.5)	7 (13.7)	4 (20.0)	
IIA	24 (33.8)	18 (35.3)	6 (30.0)	
IIB	6 (8.5)	4 (7.8)	2 (10.0)	
IIIA	5 (7.0)	4 (7.8)	1 (5.0)	
IIIB	1 (1.4)	1 (2.0)	0 (0.0)	

Values are expressed as the mean ± SD or as a number (percentage). The *p*-values compare cardiotoxicity with no cardiotoxicity. BMI: body mass index; AH: arterial hypertension; CVD: cardiovascular disease; family history of CVD: family history of premature atherosclerotic cardiovascular disease; ACE inhibitors: angiotensin-converting enzyme inhibitors; ARB: angiotensin receptor blocker; β-blocker: beta blocker; NT pro-BNP: N-terminal pro B-type natriuretic peptide; pTNM: pathological tumor-node-metastasis.

**Table 2:** Distribution of chemotherapy regimens.

Regimen	All Patients (*n* = 71)	Noncardiotoxicity (*n* = 51, 71.8%)	Cardiotoxicity (*n* = 20, 28.2%)	*p*-Value
AC	7 (9.9)	5 (9.8)	2 (10.0)	1.000
AC-paclitaxel	47 (66.2)	34 (66.7)	13 (65.0)	1.000
AC-docetaxel	6 (8.5)	5 (9.8)	1 (5.0)	0.668
FAC-docetaxel	4 (5.6)	3 (5.9)	1 (5.0)	1.000
TAC	4 (5.6)	1 (2.0)	3 (15.0)	0.065
FAC	3 (4.2)	3 (5.9)	0 (0.0)	0.554
Doxorubicin cumulative dose (mg/m^2^)	231.74 ± 29.03	231.81 ± 30.04	231.57 ± 27.01	0.505

The *p*-values compare cardiotoxicity with no cardiotoxicity. A: doxorubicin; C: cyclophosphamide; F: 5-fluorouracil; T: docetaxel.

**Table 3:** Echocardiographic parameters in baseline and follow-up results.

	Baseline (*n* = 71)	Follow-up (*n* = 71)	*p*-Value
LVEF (%)	60.51 ± 1.89	53.94 ± 2.83	<0.001
GLS (%)	−20.96 ± 0.51	−18.02 ± 1.01	<0.001
LVEDD (mm)	46.25 ± 3.85	47.11 ± 3.76	0.157
LVEDD index (mm/m^2^)	24.94 ± 2.82	25.64 ± 3.69	0.204
MAPSE (mm)	14.96 ± 1.89	13.32 ± 1.74	<0.001
S′ mean (cm/s)	9.17 ± 1.35	8.07 ± 1.21	<0.001
E (cm/s)	73.38 ± 15.15	67.0 ± 13.14	<0.022
A (cm/s)	72.49 ± 17.52	77.49 ± 18.07	0.126
E/A ratio	1.08 ± 0.36	0.91 ± 0.28	0.003
E′ mean (cm/s)	11.58 ± 2.83	9.94 ± 2.27	<0.001
E/e′	6.65 ± 1.39	7.17 ± 1.53	0.037

Values are expressed as the mean ± SD. The *p*-values compare cardiotoxicity with no cardiotoxicity. LVEF: left ventricular ejection fraction; GLS: global longitudinal strain; LVEDD: left ventricular end-diastolic diameter; LVEDDi: left ventricular end-diastolic diameter index; MAPSE: mitral annular plane systolic excursion; S: mitral annular plane peak systolic velocity average, E: peak E-wave velocity, A: peak A-wave velocity, E′: global peak mitral annular velocity during early filling.

**Table 4:** 2D echocardiographic parameters before and after chemotherapy in patients with and without cardiotoxicity.

Variables	Cardiotoxicity (*n* = 20, 28.2%)			No Cardiotoxicity (*n* = 51, 71.8%)		
	Baseline	Follow-Up	*p*-Value	Baseline	Follow-Up	*p*-Value
LVEF (%)	62.20 ± 1.88	50.20 ± 2.38	<0.001	59.84 ± 1.43	55.41 ± 1.13	<0.001
GLS (%)	−20.85 ± 0.35	−17.03 ± 0.52	<0.001	−21.00 ± 0.55	−18.40 ± 0.88	<0.001
LVEDD (mm)	46.75 ± 2.45	47.50 ± 3.25	0.413	46.06 ± 4.28	46.95 ± 3.96	0.215
LVEDD index (mm/m^2^)	25.28 ± 3.10	25.75 ± 2.95	0.623	24.81 ± 2.72	25.60 ± 3.97	0.242
MAPSE (mm)	14.99 ± 1.69	13.01 ± 1.84	<0.001	14.95 ± 1.99	13.44 ± 1.70	<0.001
S′ mean (cm/s)	9.02 ± 1.18	7.62 ± 1.17	<0.001	9.23 ± 1.42	8.24 ± 1.19	<0.001
E (cm/s)	73.70 ± 15.18	65.85 ± 11.74	0.107	73.25 ± 15.28	67.45 ± 13.73	0.079
A (cm/s)	70.15 ± 17.35	76.95 ± 19.16	0.247	73.41 ± 17.66	77.71 ± 17.82	0.225
E/A ratio	1.12 ± 0.37	0.91 ± 0.32	0.022	1.07 ± 0.36	0.90 ± 0.26	0.019
E′ mean (cm/s)	11.41 ± 2.38	9.99 ± 2.61	0.082	11.64 ± 2.40	9.92 ± 2.15	<0.001
E/E′ ratio	6.77 ± 1.24	7.20 ± 1.32	0.291	6.60 ± 1.46	7.15 ± 1.62	0.066

Values are expressed as the mean ± SD. LVEF: left ventricular ejection fraction; GLS: global longitudinal strain; LVEDD: left ventricular end-diastolic diameter; MAPSE: mitral annular plane systolic excursion; S′: mitral annular peak systolic velocity, E′: peak early diastolic transmitral flow velocity; A: peak late (atrial) diastolic transmitral flow velocity; E′: peak mitral annular tissue velocity during early filling; E/E: ratio of peak early diastolic transmitral flow velocity to peak early mitral annular tissue velocity.

**Table 5:** Associations between polymorphisms and cardiotoxicity.

Polymorphism	Cardiotoxicity		*p*-Value *	OR (95% CI)	*p*-Value	Adjusted OR ^a^ (95% CI)	*p*-Value	Adjusted OR ^b^ (95% CI)	*p*-Value
	No (*n* = 51, 71.8%)	Yes (*n* = 20, 28.2%)							
*ABCB1* rs1045642									
TT	10 (19.6)	5 (25.0)	0.806	1 (reference)		1 (reference)		1 (reference)	
CT	33 (64.7)	11 (55.0)		0.667 (0.187–2.379)	0.532	0.640 (0.146–2.806)	0.554	0.603 (0.136–2.672)	0.505
CC	8 (15.7)	4 (20.0)		1.000 (0.200–5.004)	1.000	1.235 (0.199–7.665)	0.821	1.319 (0.209–8.310)	0.768
CC, CT vs. TT				0.732 (0.215–2.492)	0.617	0.752 (0.182–3.102)	0.693	0.737 (0.178–3.047)	0.673
CC vs. CT, TT				1.344 (0.355–5.083)	0.663	1.704 (0.383–7.589)	0.484	1.870 (0.401–8.725)	0.426
*ABCC1* rs4148350									
GG	48 (94.1)	15 (75.0)		1 (reference)		1 (reference)		1 (reference)	
TG	2 (3.9)	5 (25.0)	**0.016**	8.000 (1.405–45.547)	**0.019**	9.258 (1.364–62.846)	**0.023**	9.661 (1.418–65.824)	**0.021**
TT	1 (2.0)	0 (0.0)		x		x		x	
TT, TG vs. GG				5.333 (1.138–24.985)	**0.034**	6.370 (1.171–34.642)	0.032	6.544 (1.202–35.615)	**0.030**
TT vs. TG, GG				x		x		x	
*ABCC1* rs3743527									
CC	29 (56.9)	12 (60.0)	0.856	1 (reference)		1 (reference)		1 (reference)	
CT	17 (33.3)	7 (35.0)		0.995 (0.329–3.013)	0.993	0.846 (0.224–3.196)	0.805	0.834 (0.220–3.169)	0.790
TT	5 (9.8)	1 (5.0)		0.483 (0.051–4.586)	0.527	0.638 (0.061–6.707)	0.708	0.626 (0.060–6.582)	0.697
TT, CT vs. CC				0.879 (0.307–2.517)	0.810	0.797 (0.234–2.709)	0.716	0.784 (0.229–2.680)	0.698
TT vs. CT, CC				0.484 (0.053–4.425)	0.521	0.669 (0.066–6.796)	0.734	0.660 (0.065–6.696)	0.725

* Chi-square test. ^a^ Adjusted for GLS and NT-proBNP. ^b^ Adjusted for GLS, NT-proBNP, and the cumulative DOX dose. Significant *p*-values are marked in bold. OR: odds ratio; CI: confidence interval; “x”: not applicable.

## Data Availability

The data presented in this study are available on request from the corresponding author.
